# Integrative Analysis of Three Novel Competing Endogenous RNA Biomarkers with a Prognostic Value in Lung Adenocarcinoma

**DOI:** 10.1155/2020/2837906

**Published:** 2020-08-04

**Authors:** Juan Tan, Weimin Wang, Bin Song, Yingjian Song, Zili Meng

**Affiliations:** ^1^Department of Gerontology, The Affiliated Huaian No. 1 People's Hospital of Nanjing Medical University, Huai'an, Jiangsu, China; ^2^Department of Endocrinology, Clinical Medical College of Yangzhou University, Yangzhou, Jiangsu, China; ^3^Department of Respiratory Medicine, The Affiliated Huaian No. 1 People's Hospital of Nanjing Medical University, Huai'an, Jiangsu, China

## Abstract

Increasing evidence has shown competitive endogenous RNAs (ceRNAs) play key roles in numerous cancers. Nevertheless, the ceRNA network that can predict the prognosis of lung adenocarcinoma (LUAD) is still lacking. The aim of the present study was to identify the prognostic value of key ceRNAs in lung tumorigenesis. Differentially expressed (DE) RNAs were identified between LUAD and adjacent normal samples by limma package in R using The Cancer Genome Atlas database (TCGA). Gene ontology (GO) and Kyoto Encyclopedia of Genes and Genomes (KEGG) pathway function enrichment analysis was performed using the clusterProfiler package in R. Subsequently, the LUAD ceRNA network was established in three steps based on ceRNA hypothesis. Hub RNAs were identified using degree analysis methods based on Cytoscape plugin cytoHubba. Multivariate Cox regression analysis was implemented to calculate the risk score using the candidate ceRNAs and overall survival information. The survival differences between the high-risk and low-risk ceRNA groups were determined by the Kaplan-Meier and log-rank test using survival and survminer package in R. A total of 2,989 mRNAs, 185 lncRNAs, and 153 miRNAs were identified. GO and KEGG pathway function enrichment analysis showed that DE mRNAs were mainly associated with “sister chromatid segregation,” “regulation of angiogenesis,” “cell adhesion molecules (CAMs),” “cell cycle,” and “ECM-receptor interaction.” LUAD-related ceRNA network was constructed, which comprised of 54 nodes and 78 edges. Top ten hub RNAs (hsa-miR-374a-5p, hsa-miR-374b-5p, hsa-miR-340-5p, hsa-miR-377-3p, hsa-miR-21-5p, hsa-miR-326, SNHG1, RALGPS2, and PITX2) were identified according to their degree. Kaplan-Meier survival analyses demonstrated that hsa-miR-21-5p and RALGPS2 had a significant prognostic value. Finally, we found that a high risk of three novel ceRNA interactions (SNHG1-hsa-miR-21-5p-RALGPS2, SNHG1-hsa-miR-326-RALGPS2, and SNHG1-hsa-miR-377-3p-RALGPS2) was positively associated with worse prognosis. Three novel ceRNAs (SNHG1-hsa-miR-21-5p-RALGPS2, SNHG1-hsa-miR-326-RALGPS2, and SNHG1-hsa-miR-377-3p-RALGPS2) might be potential biomarkers for the prognosis and treatment of LUAD.

## 1. Introduction

Although incidence and mortality have declined, lung cancer remains the leading cause of cancer-related death in both men and women in the United States, representing about 23% of all cancer deaths [1]. Lung adenocarcinoma (LUAD) is the most commonly diagnosed histological subtype in nonsmoking people. It accounts for about 40% of all lung cancer cases [2]. Since the earliest stages of LUAD are often asymptomatic, the majority of patients are diagnosed at advanced cancer stages. Patients following surgical resection can frequently present with local or distant tumor recurrence. The primary risk factor for lung adenocarcinoma is smoking, but approximately 10-15% of cases occur in never smokers [3]. This past decade has witnessed driver mutations of epidermal growth factor receptor (EGFR), Kirsten Rat Sarcoma Viral Protooncogene (KRAS), and anaplastic lymphoma kinase (ALK) rearrangements in LUAD [4]. Developments in the characterization of lung cancer genomic alterations, molecularly targeted therapies have dramatically improved outcomes in a subset of patients [5]. However, most patients with metastatic adenocarcinoma are treated with conventional chemotherapy attributed to lacking an identifiable driver oncogene [6]. Despite advances in surgery, chemotherapy, radiation therapy, and new targeted therapy for non-small-cell lung cancer, the 5-year survival rate is only about 16% [7]. Therefore, the identification of underlying molecular mechanisms and novel prognostic biomarkers in LUAD should enable the development of more effective diagnostic and therapeutic strategies.

The concept of competitive endogenous RNA (ceRNA) was first presented by Salmena and colleagues in 2011 [8]. In theory, ceRNAs are comprised of transcripts that can competitively bind to common miRNAs leading to regulate target gene expression [9]. All RNAs sharing MREs can form a deregulate ceRNA network contributing to the initiation and progression of human cancer [10]. CeRNAs can connect protein-coding mRNAs to noncoding RNAs such as microRNA, long noncoding RNA, pseudogenic RNA, and circular RNA at the posttranscription level. Increasing evidence has shown that miRNA-mediated ceRNA regulatory mechanisms play critical roles in various pathological processes, including numerous cancers. A recent study revealed that the MMP9/ITGB1-miR-29b-3p-HCP5 subnetwork was linked to the prognosis of pancreatic cancer [11]. In addition, another recent study reported four lncRNA-based ceRNA (ADAMTS9-AS1, LINC00536, AL391421.1, and LINC00491) had a significant prognostic value in breast cancer [12]. Collectively, these findings document that dysregulation of the lncRNA-miRNA-mRNA network contributes to the pathogenesis of various cancers. Nevertheless, current information on the lncRNA-miRNA-mRNA network in LUAD is not enough.

In the present study, we aimed to construct a ceRNA network using The Cancer Genome Atlas database- (TCGA-) LUAD dataset. Further, we examined the relationship between the identified genes and ceRNA interaction modules with overall survival and prognosis. Through our study, we sought to gain new insights into the molecular mechanisms underlying LUAD and identify potential biomarkers in the prognosis of this disease.

## 2. Materials and Methods

### 2.1. Materials

#### 2.1.1. Data Source and Preprocessing

Lung adenocarcinoma RNA-seq and miRNA-seq expression data were obtained from The Cancer Genome Atlas database (TCGA, https://portal.gdc.cancer.gov/). We collected data according to the following steps: (1) The project name was TCGA-LUAD. (2) The disease type was adenocarcinomas. (3) The selected samples had no other malignancies. RNA-seq expression data consisted of 495 LUAD tissue samples and 54 adjacent normal samples. miRNA-seq expression data included 481 LUAD tissue samples and 45 adjacent normal samples.

### 2.2. Methods

#### 2.2.1. Screening of Differentially Expressed (DE) RNAs

The RNA and miRNA raw sequencing read counts were normalized using the voom package provided by R [13]. The limma package in R was used to identify the differentially expressed mRNAs, lncRNAs, and miRNAs between lung adenocarcinoma samples and adjacent samples [14]. An adjusted *P* value (adj. *P*) < 0.01 and ∣logFC | >1 were set as the cut-off criteria. Volcano plots were visualized using the ggplot2 packages in R. The heat map was plotted using the heatmap.2 package in R.

#### 2.2.2. Functional and Pathway Enrichment Analysis

Gene ontology (GO) and Kyoto Encyclopedia of Genes and Genomes (KEGG) pathway function enrichment analysis of DE mRNAs was performed using the clusterProfiler package in R [15]. Pathways with *q* value < 0.01 were defined as significantly enriched, with *P* value < 0.05 as the cut-off criterion.

#### 2.2.3. Construction of the LUAD ceRNA Network

The LUAD ceRNA network was established in three steps based on the ceRNA hypothesis: (1) By starBase v2.0 matching, candidate miRNA-lncRNA and miRNA-mRNA interactions were required to share significantly more common miRNAs. The spongeScan was used to predict binding sites of lncRNA and miRNA. A hypergeometric test was used to examine each of the potential ceRNA pairs. *P* values < 0.05 was set as a cut-off criterion of significant ceRNA pairs. (2) To determine the positive regulation between DE lncRNAs and DE mRNAs, Pearson's correlation coefficient (PCC) was calculated. We selected *P* < 0.05 as a threshold. (3) According to the ceRNA theory, the candidate miRNA-mRNA and miRNA-lncRNA interactions should meet in a similar regulation mode. Two methods were used to evaluate miRNA regulation mode. The regulatory similarity score was defined to assess the similarity between miRNA-lncRNA and miRNA-mRNA interactions. The regulation similarity score was computed as follows:

(1) $$\begin{array}{c} \text{Regulation similarity score}=1-\frac{1}{M}\overset{M}{\underset{k=1}{\sum }}{\left[\frac{\left|\text{corr}\left(m_{k},l\right)-\text{corr}\left(m_{k},g\right)\right|}{\;|\;\text{corr}\left(m_{k},l\right)\;|\;+\;|\;\text{corr}\left(m_{k},g\right)\;|\;}\right]}^{M}. \end{array}$$

In this formulation, *M* is the total number of shared miRNAs that interact with the DE lncRNAs and mRNAs; *K* is the *k*th shared miRNA; corr(*m*_*k*_, *l*) represents Pearson's correlation coefficient between the *k*th shared miRNA and lncRNA. corr(*m*_*k*_, *l*) represents Pearson's correlation coefficient between the *k*th shared miRNA and mRNA. The higher the regulation similarity score, the more significant this ceRNA pair is. The criteria of reliable ceRNA pairs were regulation similarity score > 0. Through these steps, the LUAD ceRNA network was constructed and visualized using Cytoscape v3.6. Additionally, the top ten hub RNAs were identified using degree analysis methods based on Cytoscape plugin cytoHubba.

#### 2.2.4. Survival Analysis

To examine the association of hub RNA (mRNA, lncRNAs, and miRNAs) expression levels with overall survival, we performed Kaplan-Meier survival analyses with the log-rank test using the survival package in R. To explore the potential impact of candidate ceRNAs on the prognostic survival, multivariate Cox regression analysis was implemented to calculate the risk score (RS) using the candidate ceRNAs and overall survival information. Then, the patients were divided into the low-risk and high-risk groups according to the median risk scores. The survival differences between the high-risk and low-risk groups were determined by the log-rank test using the survival and survminer package in R (*P* < 0.05 was selected as a threshold). The risk score was calculated using the following formula:

(2) $$\begin{array}{c} \text{Risk score}=\overset{n}{\underset{i}{\sum }}\beta_{i}*x_{i}. \end{array}$$

In this formulation, *β*_*i*_ indicates the coefficients evaluated with gene expression and *x*_*i*_ refers to the gene relative expression level.

## 3. Results

### 3.1. Differentially Expressed RNAs in LUAD

**Figure 1:**
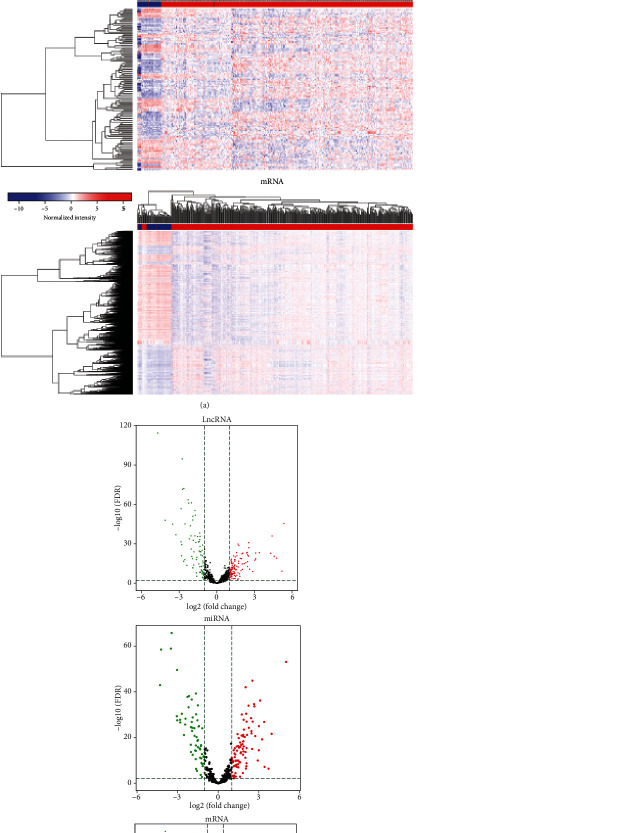
(a) Heat map plot of the overlapped DEGs (lncRNAs, miRNAs, and mRNAs) between LUAD and normal samples in dataset TCGA. Notes: red represents higher expression, and blue represents lower expression. Abbreviation: DEGs: differentially expressed genes; LUAD: lung adenocarcinoma. (b) Volcano plot showing the DEGs (lncRNAs, miRNAs, and mRNAs) between LUAD and normal samples in dataset TCGA. *X*-axis indicates the mean expression differences of genes between LUAD and normal samples, and *Y*-axis represents log-transformed *P* value. Note: the black dots represent genes that are not differentially expressed between LUAD and normal samples, and the green dots and red dots represent the downregulated and upregulated genes in LUAD samples, respectively. >1 >2 and adj. *P* value < 0.01 were set as the cut-off criteria. Abbreviation: DEGs: differentially expressed genes; LUAD: lung adenocarcinoma.

A total of 2,989 mRNAs (1132 up- and 1857 downregulated), 185 lncRNAs (106 up- and 79 downregulated), and 153 miRNAs (89 up- and 64 downregulated) differentially expressed RNAs were identified between LUAD and adjacent normal samples using the TCGA database by the R package, with *P* < 0.01 and >1 >2 as the thresholds. Volcano plots showed the distribution of the differentially expressed RNAs (mRNAs, lncRNAs, and miRNAs) (Figure 1(a)). The heat map displayed clear separation and consistency in the expression profiles of the LUAD and normal samples (Figure 1(b)).

### 3.2. Functional and Pathway Enrichment of DE mRNAs

**Figure 2:**
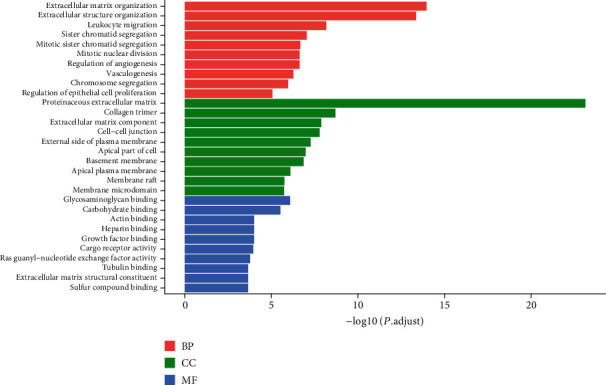
GO functional annotation for the significant DEGs between LUAD and normal samples in dataset TCGA. Red represents the top ten enriched biological processes (BP) of the DEGs, green represents the top ten enriched cellular components (CC), and blue represents the top ten enriched molecular function (MF). Abbreviation: DEGs: differentially expressed genes; LUAD: lung adenocarcinoma.

**Figure 3:**
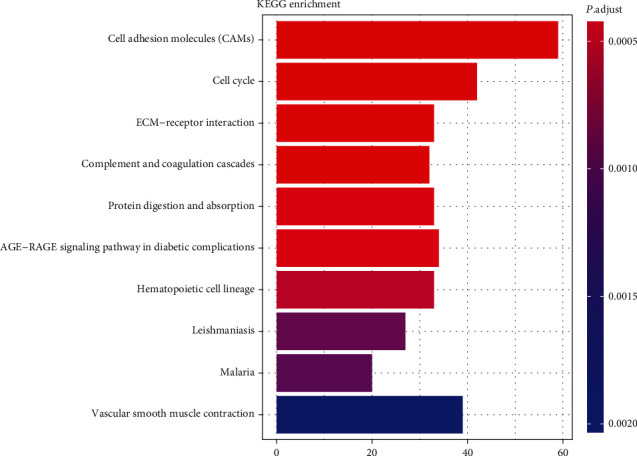
The top ten enriched KEGG pathways of the significant DEGs between LUAD and normal samples in dataset TCGA. Abbreviation: DEGs: differentially expressed genes; LUAD: lung adenocarcinoma. *X*-axis indicates the gene count.

To understand the function and mechanism of DE mRNAs, GO and KEGG enrichment analyses were analyzed with the R package. As shown in Figure 2, the important biological process terms identified by GO analysis included “sister chromatid segregation,” “mitotic sister chromatid segregation,” “mitotic nuclear division,” “regulation of angiogenesis,” and “chromosome segregation.” The most enriched KEGG pathways were “cell adhesion molecules (CAMs),” “cell cycle,” “ECM-receptor interaction,” “complement and coagulation cascades,” and “protein digestion and absorption” (Figure 3). Our analysis showed that 2,989 DE mRNAs were mainly associated with signaling pathways relevant to human tumorigenesis and metabolism processing.

### 3.3. Construction of the LUAD-Related ceRNA Network

**Figure 4:**
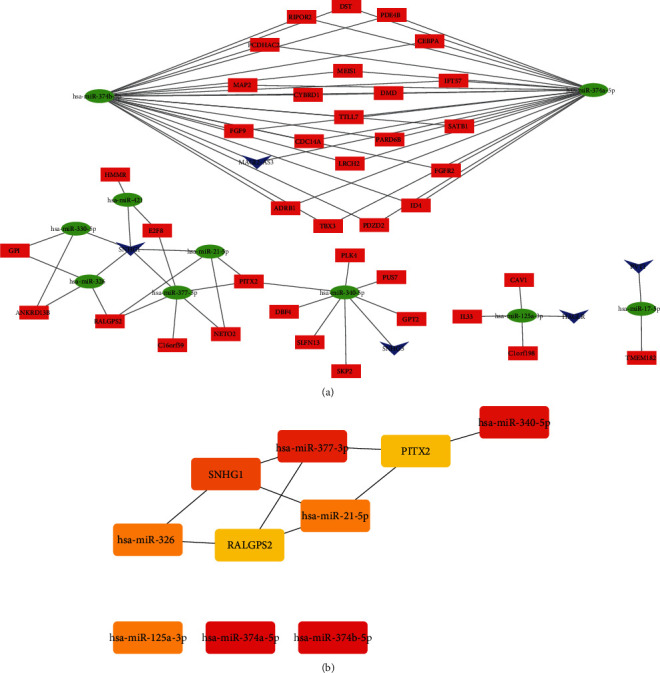
(a) lncRNA-miRNA-mRNA competing endogenous RNA (ceRNA) network constructed from DEGs. (b) Sub-ceRNA network obtained from the ceRNA network based on hub RNAs.

Based on the three steps, significant miRNA-targeted RNAs were predicted by the starBase v2.0 database, positively regulated lncRNA-mRNA interactions were assessed using PPC, and miRNA-lncRNA interactions with the miRNA-mRNA interactions were combined based on the regulation similarity score. Finally, the LUAD-related lncRNA-miRNA-mRNA ceRNA network was constructed (Figure 4(a)). The network comprised 54 nodes (5 lncRNAs, 39 mRNAs, and 10 miRNAs) and 78 edges. Top ten hub RNAs were identified according to their degree by cytoHubba, consisting of hsa-miR-374a-5p, hsa-miR-374b-5p, hsa-miR-340-5p, hsa-miR-377-3p, hsa-miR-125a-3p, hsa-miR-21-5p, hsa-miR-326, SNHG1, RALGPS2, and PITX2. As shown in Figure 4(b), a significant sub-ceRNA network was obtained from the LUAD-related network based on hub RNAs, including 10 nodes and 9 edges. Finally, we found five master lncRNA-miRNA-mRNA interactions (SNHG1-hsa-miR-21-5p-RALGPS2, SNHG1-hsa-miR-21-5p-PITX2, SNHG1-hsa-miR-326-RALGPS2, SNHG1-hsa-miR-377-3p-PITX2, and SNHG1-hsa-miR-377-3p-RALGPS2) may play very important roles in modulation of initiation and progression of LUAD.

### 3.4. Prognosis Analysis of Key ceRNA Networks

**Figure 5:**
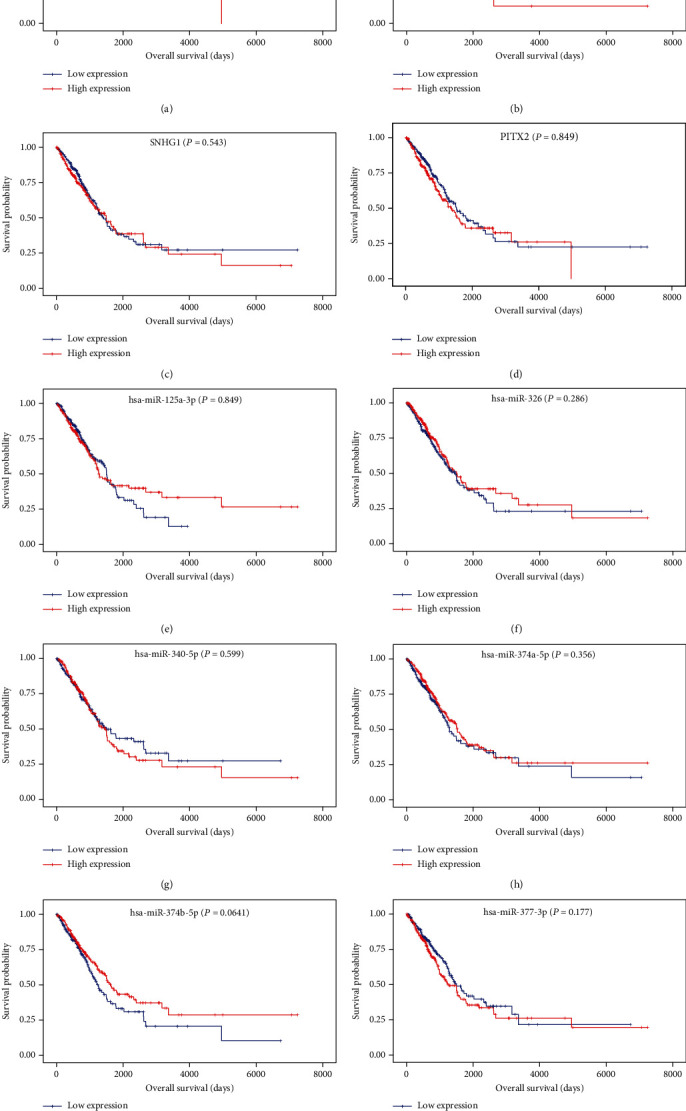
Kaplan-Meier survival curves for 10 hub RNAs (1lncRNA, 7 miRNAs, and 2 mRNAs) in the ceRNA network (horizontal axis: overall survival times: days, vertical axis: survival function). High expression of RALGPS2 (a) and hsa-miR-21-5p (b) was associated with a low proportion of overall survival (*P* = 0.0052 and 0.0372, respectively); no significant associations were observed between survival and other eight hub RNAs.

**Figure 6:**
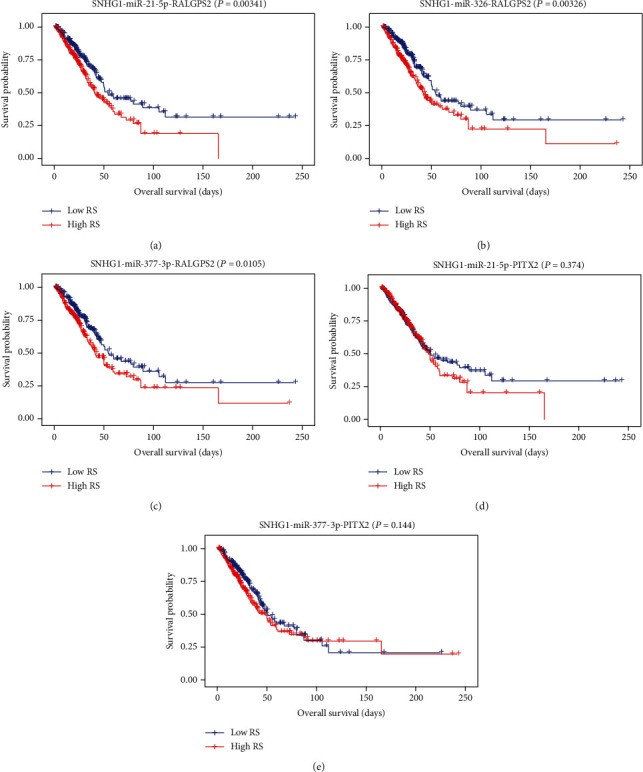
Kaplan-Meier survival curves for five ceRNA pairs associated with overall survival (horizontal axis: overall survival times: days, vertical axis: survival function). High-risk score of SNHG1-hsa-miR-21-5p-RALGPS2 (a), SNHG1-hsa-miR-326-RALGPS2 (b), and SNHG1-hsa-miR-377-3p-RALGPS2 (c) was associated with low proportion of overall survival (*P* = 0.00341, 0.00326, and 0.0105, respectively); no significant associations were observed between survival and SNHG1-hsa-miR-21-5p-PITX2 (d) and SNHG1-hsa-miR-377-3p-PITX2 (e).

To examine the potential impact of the expression level of hub RNAs (mRNA, lncRNAs, and miRNAs) on the prognostic survival of LUAD patients, Kaplan-Meier survival analyses were performed. As shown in Figure 5, we detected high expression of hsa-miR-21-5p, and RALGPS2 were positively significantly associated with worse survival status. However, no significant associations were observed between survival and other eight hub RNAs. To further explore the key role of lncRNA-miRNA-mRNA interactions in the initiation and progression of LUAD, we calculated the risk score of each ceRNA to determine the prognostic-significant lncRNA-miRNA-mRNA interactions. As presented in Figure 6, Kaplan-Meier survival curves indicated SNHG1-hsa-miR-21-5p-RALGPS2, SNHG1-hsa-miR-326-RALGPS2, and SNHG1-hsa-miR-377-3p-RALGPS2 were positively associated with overall survival time, whereas there was no significant association of SNHG1-hsa-miR-21-5p-PITX2 and SNHG1-hsa-miR-377-3p-PITX2 with survival.

## 4. Discussion

In 2019, lung cancer accounted for about 13% of all new cancers and 24% of all cancer deaths in the USA, with an estimated 228,820 new cases and 135,720 deaths 1. The current high mortality in LUAD patients may be attributed to short of specific diagnostic and prognostic biomarkers. Therefore, it is essential to investigate LUAD-related underlying molecular mechanisms and potential prognostic indicators. Accumulating evidence has documented that dysregulated ceRNAs are associated with cancer initiation and progression [16]. Numerous studies are focusing on them as they can offer novel insights into cancer pathogenesis and exploration of more effective treatments.

In recent years, some studies have revealed deregulate ceRNAs in LUAD. For example, the previous study constructed the LUAD-related ceRNA networks based on common miRNAs and differentially expressed RNAs as well as coexpression, but did not include similar regulation mode among ceRNA pairs [17]. Another study also established a non-small-cell lung cancer-specific ceRNA network to explore lncRNAs associated with the survival of patients. Still, they did not consider the relationship between survival and ceRNAs nor construct the prognostic signature [18]. In the present study, we constructed LUAD-related ceRNAs in three steps based on the ceRNA theory. Furthermore, we revealed three novel lncRNA-miRNA-mRNA prognostic signatures involved in tumorigenesis and progression of LUAD.

In detail, a total of 3,327 DE RNAs were identified between LUAD and normal lung tissues from the TCGA database, comprising of 2,989 mRNAs, 185 lncRNAs, and 153 miRNAs. GO analysis showed differentially expressed genes were mainly enriched in “sister chromatid segregation,” “mitotic sister chromatid segregation,” “mitotic nuclear division,” “regulation of angiogenesis,” and “chromosome segregation.” Besides, the significant KEGG pathways were “cell adhesion molecules,” “cell cycle,” “ECM-receptor interaction,” “complement and coagulation cascades,” and “protein digestion and absorption.” It has been well established that adhesion molecules, cell cycle, and ECM-receptor interaction play very important roles in cancer initiation and progression [19–21]. Therefore, these significant differentially expressed genes may be involved in promoting lung tumorigenesis and metastasis. Furthermore, in order to identify underlying molecular mechanisms of these significant differentially expressed genes, LUAD-related ceRNA networks were constructed. Finally, we focus on three prognostic-significant ceRNAs (SNHG1-hsa-miR-21-5p-RALGPS2, SNHG1-hsa-miR-326-RALGPS2, and SNHG1-hsa-miR-377-3p-RALGPS2) involved in occurrence and development of LUAD.

It has been reported that one miRNA can modulate multiple target RNAs that contain similar MRE. Likewise, one RNA that contain multiple MREs is under the modulation of multiple miRNAs [22]. In the present study, we detected that the three ceRNA interactions comprised of one lncRNA, three miRNAs, and one mRNA. Therefore, SNHG1 and RALGPS2 played important roles in three prognostic-significant ceRNAs. SNHG1, small nucleolar RNA host gene 1, is a host to 8 small nucleolar RNAs and locates on 11q12.3 with 11 exons [23]. Accumulating evidence showed that dysregulation of SNHG1 played crucial roles in numerous human cancers [24–27]. In addition, previous studies showed that SNHG1 could function as ceRNA, playing a vital role in cancer development. In a study by Lu et al., SNHG1 functioned as a ceRNA for miR-145-5p increased the expression of MTDH and finally promoted cell proliferation, migration, and invasion in non-small-cell lung cancer. Their findings suggested that the SNHG1/miR-145-5p/MTDH axis played an important role in NSCLC and could serve as a therapeutic and diagnostic marker [28]. Similarly, Xu et al. showed that SNHG1 served as a sponge for miR-154-5p, and it promoted cell growth and proliferation and expression of CCND2 in colorectal cancer cells [29]. Furthermore, Zhang et al. documented SNHG1 was associated with prognostic survival of lung squamous cell carcinoma [30]. However, aberrant expression of SNHG1 had no impact on survival in the present study. One plausible explanation is that the prognostic value of SNHG1 might be lung cancer histotype dependent. Otherwise, it remains to be demonstrated in a future study of whether SNHG1 could display a favorable prognostic role in LUAD. In our study, some lncRNA-miRNA interactions have been reported. For instance, Wang et al. revealed that miR-326 inhibited cell growth, migration, and invasion in osteosarcoma by targeting SNHG1 [31]. These reports further demonstrate the accuracy of our present analytic results. Some other miRNAs, such as miR-382-5p, miR-199-3p, and miR-195, have also been suggested as the target of SNHG1 [32–34], which likely also play important parts in human cancer. To the best of our knowledge, we first revealed SNHG1 could act as a ceRNA of regulating the expression of RALGPS2 by sponging miR-21-5p and miR-377-3p. A significant prognostic survival was observed in RALGPS2 and hsa-miR-21-5p, but not in SNHG1 and miR-377-3p. In agreement with our findings, dysregulated miR-21-5p was considered as an important prognostic biomarker for the overall survival of NSCLC patients [35]. Inspiringly, three novel ceRNA interactions were identified and presented a significant prognostic value in LUAD. Besides, RALGPS2, Ral guanine nucleotide exchange factor with PH domain and SH3 domain-binding motif 2, is a Ras-independent guanine nucleotide exchange factor (GEF) for Ras-related protein Ral-A (RalA) GTPase containing a PH domain and an SH3-binding region, and it is involved in cytokinesis, cell cycle, and cytoskeleton rearrangement [36]. Although RalA and RalGEFs are implicated in tumorigenesis, there are few data for RalGPS2 role in lung cancer [37]. Santos et al. showed that RALGPS2 is involved in the control of cell cycle progression in NSCLC cell lines but did not include its association with clinical features [38]. We identified the expression levels of RALGPS2 were significantly higher in LUAD tissues than adjacent normal tissues, which supported the hypothesis that RALGPS2 could be a valuable diagnostic marker for LUAD. We further revealed RALGPS2 was associated with the survival of LUAD patients. Taken together, RALGPS2 might act as an oncogene that promoted LUAD tumorigenesis and development via three ceRNA networks (SNHG1-hsa-miR-21-5p-RALGPS2, SNHG1-hsa-miR-326-RALGPS2, and SNHG1-hsa-miR-377-3p-RALGPS2).

## 5. Conclusion

In the present study, we identified three novel ceRNA networks (SNHG1-hsa-miR-21-5p-RALGPS2, SNHG1-hsa-miR-326-RALGPS2, and SNHG1-hsa-miR-377-3p-RALGPS2) with a prognostic value involved in LUAD. These ceRNA networks could potentially be involved in lung cancer development. Nevertheless, further investigations are required to establish the mechanisms of these genes in LUAD.

## Data Availability

The datasets used and analyzed during the current study are available from the corresponding author on reasonable request.
